# Guidelines for measuring and reporting particle removal efficiency in fibrous media

**DOI:** 10.1038/s41467-023-41154-4

**Published:** 2023-09-01

**Authors:** Paolo Tronville, Vincenzo Gentile, Jesus Marval

**Affiliations:** 1 Department of Energy, Politecnico di Torino, Corso Duca degli Abruzzi 24, Turin, Italy; 2 Air Quality and Aerosol Metrology Group, Atmospheric Environmental Science Department, National Physical Laboratory (NPL), Hampton Road, Teddington, TW11 0LW United Kingdom

**Keywords:** Characterization and analytical techniques, Chemical engineering, Pollution remediation, Biomedical engineering, Applied physics

## Abstract

Adopting standardized and reliable methodologies to accurately measure particle removal efficiency when developing fibrous materials for controlling airborne contamination is crucial. Here, the authors recommend best practices for experimental assessments and reporting to ensure a reliable evaluation of new airborne particle filtration media and technologies.

## Teaser

Adopting standardized and reliable methodologies to accurately measure particle removal efficiency when developing fibrous materials for controlling airborne contamination is crucial. Here, we introduce well-established aerosol science and technology concepts often overlooked or misused in interdisciplinary studies developing new fibrous materials for particle filtration. We recommend best practices for experimental assessments and reporting to ensure a reliable evaluation of new airborne particle filtration media and technologies.

## The origin of the term “PM” and its definition according to the US Code of Federal Regulations

Despite the growing concern about the health effects of exposure to airborne particles smaller than 1 μm, especially to those below 100 nm^1–3^, no widely recognized or established air quality standards define PM_1_ or PM_0.1_. The mass fraction of atmospheric particulate matter (PM) below 2.5 μm is composed mainly of particles smaller than 1 μm^4^, especially for urban aerosols. Fibrous filters performing well between the most penetrating particle size (MPPS) and 1 μm will also exhibit high efficiency below 100 nm due to the Brownian diffusion effect, which enhances particle capture efficiency as particle size decreases^5^. Hence, particle sizes between the MPPS and 1 μm represent the technological challenge for material scientists developing new fibrous filter media. The absence of air quality standards below 1 μm and 100 nm is a challenge for safeguarding public health from airborne PM.

US Federal Reference Method (FRM) 40 CFR Appendix J and L to Part 50^6–8^ and European Standard EN 12341^9^ define PM_10_ and PM_2.5_ as the PM penetrating a size-selective inlet with 50% efficiency at 10 μm and 2.5 μm aerodynamic diameters, respectively. The aerodynamic diameter is the diameter of a spherical particle with a density of 1000 kg m^−3^ with the same settling velocity (due to the force of gravity) as the measured particle. A particle having a specific aerodynamic diameter behaves aerodynamically like a water droplet with that diameter, regardless of its shape, density, or physical size^5^. Hence, the particle size defined by these methods depends on density and shape. For example, salt and iron particles having the same aerodynamic diameter will have different physical dimensions.

At the same time, the aerodynamic diameter is inappropriate to characterize smaller particles for which the force of gravity is negligible. The net transport does not depend on particle density^5^. For this reason, the physical diameter is more appropriate to characterize the behavior of particles smaller than 100 nm. Hence, a generic definition such as PM_0.1_ lacks specifications about whether the 0.1 µm size is aerodynamic or physical.

**Fig. 1:**
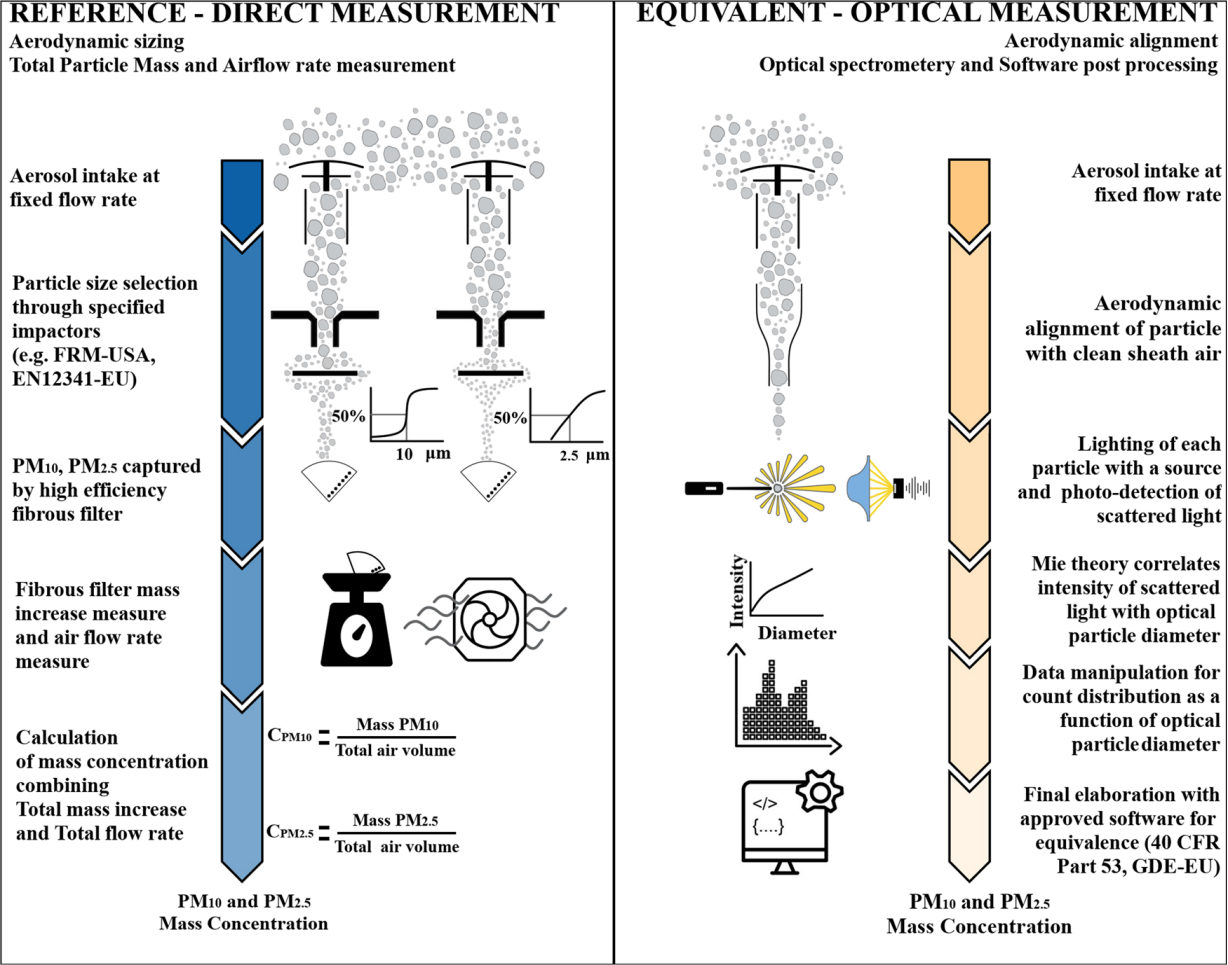
Schematization and comparison of the two methodologies used by government authorities for the environmental measurement of PM_10_ and PM_2.5_ concentrations. On the left is the schematic of the reference method, based on the direct measurement of the mass increase of a filter material collecting the aerosol. The size-selective impactor specific for PM_10_ and PM_2.5_ selects the particles according to their aerodynamic diameter with an efficiency of 50%. On the right is the equivalent and indirect method schematic. It uses the optical measurement of light scattered by each particle when exposed to a collimated light beam (Mie theory) and without selective impactors, as in the reference methodology. The indirect measurement, combined with data post-processing through the software calibrated by the instrument manufacturer, is equivalent to the direct one if the deviation of the results is within the ±25% error band.

The result of a measurement according to an FRM is a mass concentration obtained by measuring the mass increase, i.e., the mass of particles captured, of a flat piece of high-efficiency filter material exposed to a specific airflow. This measurement approach is typically called gravimetric, and the geometry of the size-selective inlet strongly influences the results, as shown in Fig. 1, which compares the reference and equivalent methods measuring approaches for PM_10_ and PM_2.5_ values. The U.S. and European monitoring regulations^7,8^ outline the formal specifications for PM_10_ and PM_2.5_ reference method samplers and their measurement. FRMs and EN 12341 include the specifications of all components and operational airflow rate, together with the drawings of the size-selective impactors. A rigorous definition by describing it with words and numbers is otherwise too difficult.

Many scientific papers^10–19^ use terms like PM_0.3_ and PM_0.1_ without defining them or assuming that they indicate the fraction of PM below a given size. This approach appears to replicate the statutory requirements to monitor ambient air that defines PM_10_ and PM_2.5_ with a similar definition (particles with an aerodynamic diameter equal to or less than 10 µm and 2.5 µm, respectively).

It is important to note that PM_10_ and PM_2.5_ rigorous definitions discussed above are not the same as the total amount of particles below 10 µm and 2.5 µm, respectively. The theory related to the operation of size-selective impactors explains the shape of the collection efficiency curve versus the particle size. An impactor is a device for selecting the particles collected on the filter material and weighed at the end of the measurement procedure. Some particles larger than the cut size will pass through the size-selective inlet. At the same time, some particles smaller than the cut size do not get through the impactor. Consequently, defining PM_0.3_ as the total number of particles below 0.3 µm may be intuitive, but it is not coherent with the definition of PM_10_ and PM_2.5_.

In addition, standard reference methods require one or more days of monitoring, and the prescribed measuring equipment is expensive. Hence, when quantifying PM to provide data with finer time resolution or cheaper devices, using a Federal Equivalent Method (FEM) is common. The 40 CFR Part 53^20^ (GDE for Europe^21^) describes how to validate FEMs, including appliances and techniques. In particular, they do not directly provide the mass concentration. Instead, they supply results calibrated against the reference methodology and respective instrumentation through an indirect measurement. The users can consider the measurement results equivalent to reference equipment when the accuracy is within ±25% over a specific period.

For example, optical spectrometers measure the number of particles as a function of their size. Using the data supplied by the optical instrument, it is possible to calculate the mass concentration of particles smaller than 2.5 μm (making assumptions about their shape and density) by integrating the area under the distribution function with an upper limit of 2.5 μm. Nevertheless, even using sophisticated instruments, we cannot consider such a result as an accurate mass concentration of PM_2.5_ according to its original definition without post-processing the measured data obtained from the optical instrument^22^.

This equivalent method does not adopt any gravimetric measurement or a sampling inlet determining the cut size of the particle distribution. Hence, the difference between the PM_2.5_ values provided by a reference method or instrument and other reported PM_2.5_ concentration values might be significant. The difference depends on the conversion between the measured data, i.e., the technology used to measure the particle concentration as a function of size and the calculated mass concentration. Under some conditions, the agreement between the two may be satisfactory. That is likely to occur if the sampled particles’ size, shape, material density, and relative humidity are close to those used for calibrating the instrument. However, the properties of the measured aerosol might deviate from the calibration assumptions. In this case, the agreement between the PM_2.5_ concentration provided by the alternative measurement method may differ considerably from the statutory value. Hence, it is crucial to calibrate optical spectrometers against gravimetric measurements using the same aerosol to be measured. Periodic recalibration is essential for maintaining reliable correlations.

## Removal efficiency by filtration of a certain PM fraction

The recent COVID-19 pandemic highlighted the importance of fibrous materials with fine fibers for controlling airborne contamination. Air filtration media should ensure high efficiency of removal of airborne particles smaller than 1 µm. Scientists worldwide and in many disciplines are developing new technologies and materials claiming filtration performance adequate to control airborne bioaerosols and beyond.

Fibrous filtration efficiency is a function of particle size and other parameters^23^. Using the mass of particles below a certain threshold to determine the overall efficiency of a filter material is ambiguous because the measured data will depend on the distribution of the mass of particles as a function of their size. In other words, the same total particle mass, corresponding to various particle size distributions, will produce different removal efficiencies, i.e., filtration efficiency test data.

Measuring the total mass concentration upstream and downstream of the filter media through a photometer or a nephelometer can cause poor repeatability of the test data within the same lab if the particle size distribution is not strictly the same (the following section discusses this issue). The reproducibility of the test data among different labs is even more challenging when the labs use test equipment with various components supplied by other manufacturers. The same problem can arise when using turnkey equipment bought on the market. Especially in these situations, the researcher shall define and control in detail the particle size distribution to get repeatable and reproducible data. Such distribution should always be the same for a good agreement among laboratories and scientists.

Aerosol technology experts use the lognormal distribution to characterize most particle size distributions. The median of the distribution of mass is called the mass median diameter (MMD) to distinguish it from the count median diameter (CMD). The geometric standard deviation (GSD) replaces the standard deviation of the normal distribution and quantifies the dispersion of this distribution. One can fully identify the distribution by knowing the aerosol is distributed according to a lognormal distribution, its MMD and GSD, and determine the aerosol’s mass distribution as a function of the particle size.

**Fig. 2:**
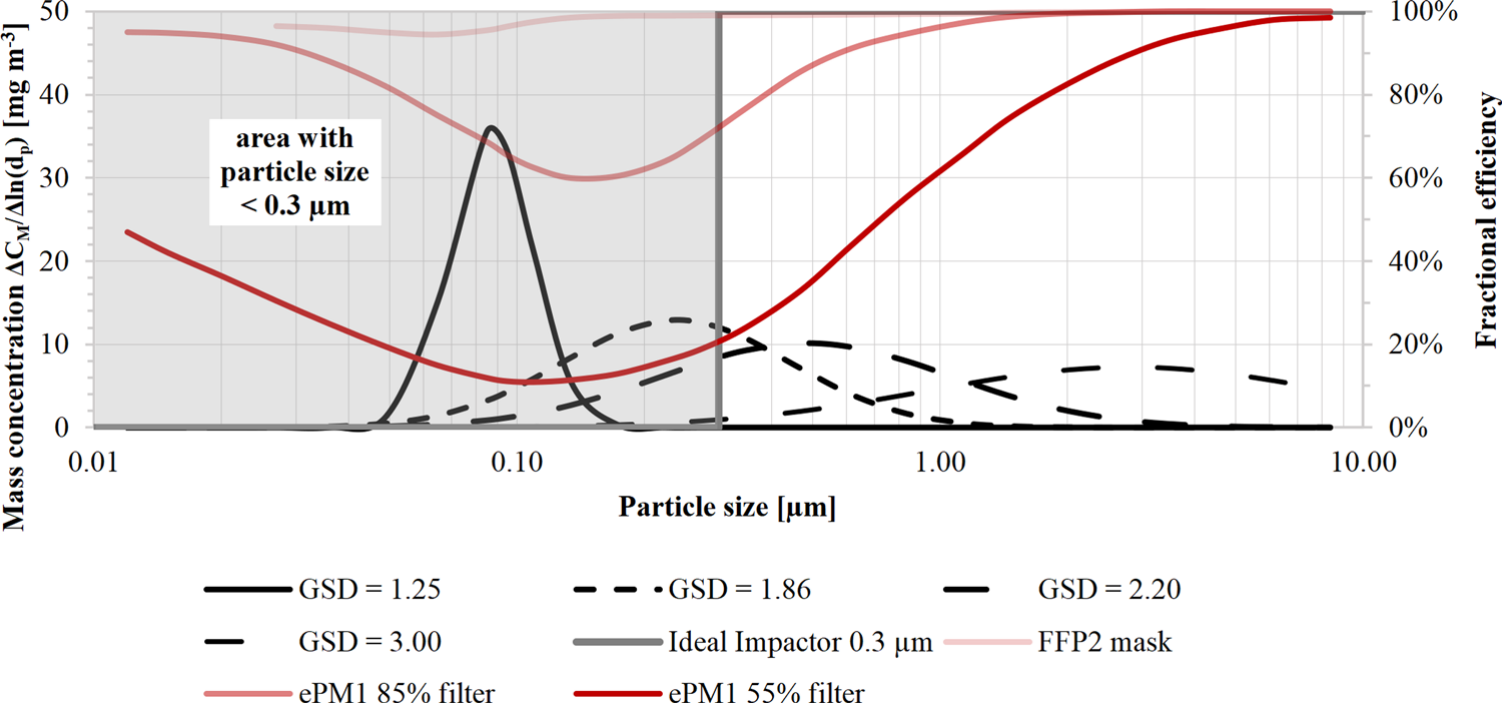
Influence of particle size distribution on filtration efficiency. The graph shows the particle size distributions of the mass concentration with CMD equal to 75 nm with four different GSDs (black lines have a GSD equal to 1.25, 1.86, 2.2, and 3, respectively). The distribution of mass concentration as a function of the particle size is lognormal. The mass concentration fraction is Δ*C*_*M*_/Δln(*d*_*p*_), where *d*_*p*_ is the particle diameter. The gray area on the left represents all the particles with an equivalent size smaller than 0.3 µm. The red lines represent typical fractional efficiencies of FFP2 mask^27^, filter *e*PM_1_ 85%^28^, and *e*PM_1_ 55%^28^.

The example in Fig. 2 shows how different particle distributions can generate different efficiency test results using a lognormal particle size distribution. All black curves show the mass distribution as a function of the particle size of an aerosol characterized by identical CMD (75 nm, the mean value prescribed by 42 C.F.R. § 84.1^24^), and total particle mass concentration (20 mg m^−3^). The different GSDs affect the shape of the particle distribution. In particular, the GSD varies from 1.25 (quasi-monodisperse aerosol) to 3.0 (polydisperse aerosol). This range is coherent with different applications of filter media. Indeed, a GSD equal to 1.25 is considered by ISO 29463-2:2011^25^ as a monodisperse aerosol for testing HEPA filters. 42 C.F.R. § 84.1 prescribes a GSD below 1.86 for the N95 labeling. EN 13274-7:2019^26^ specifies maximum GSD values of 2.2 and 3 when testing respiratory protective devices with paraffin oil and NaCl, respectively.

Figure 2 also depicts the efficiency by the particle size of three typical filter media, FFP2^27^, *e*PM_1_ 85%^28^, and *e*PM_1_ 55%^28^. Once the filter efficiency by particle size is known (red curves in Fig. 2), we can calculate the overall mass efficiency (OME) across the entire particle size range using the equation:

1 $${{{{{\rm{OME}}}}}}=\frac{\mathop{\sum }\nolimits_{i=1}^{n}{E}_{i}\cdot {q}_{m,i}\cdot \Delta {{{{{\rm{ln}}}}}}\bar{{d}_{i}}}{\mathop{\sum }\nolimits_{i=1}^{n}{q}_{m,i}\cdot \Delta {{{{{\rm{ln}}}}}}\bar{{d}_{i}}}$$

where *i* = *i*th channel, *n* = total number of channels*, E*_*i*_ = efficiency at the *i*th channel*, q*_*m,i*_ = mass concentration at the *i*th channel, $\bar{{d}_{i}}$ = geometric mean of *i*th channel.

**Fig. 3:**
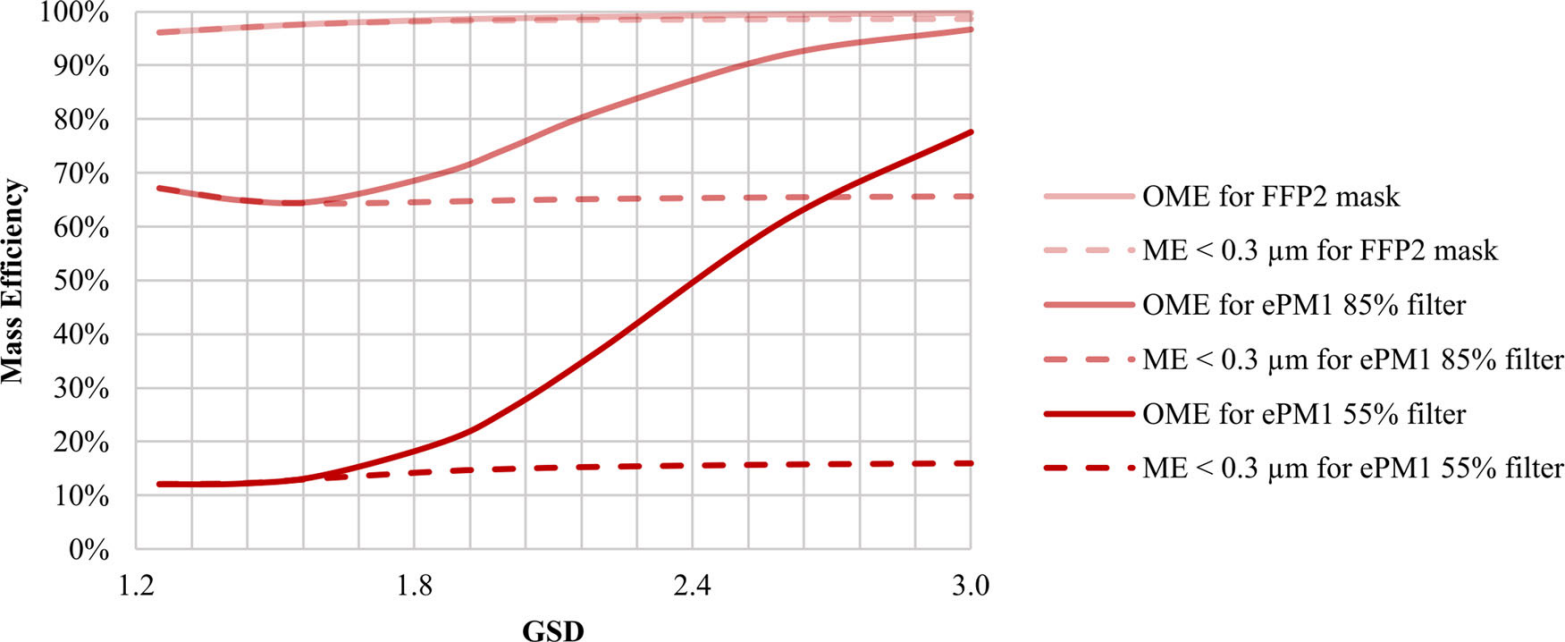
Variation of the mass efficiency for the different filter media and varying the GSD. The graph shows the efficiency differences. Note the impact of considering the entire size distribution (OME) versus only the fraction below 0.3 µm (ME < 0.3 µm) of a polydisperse test aerosol. Increasing the aerosol dispersion (e.g., increasing GSD from 1.2 to 3) results in a significant difference between OME and ME. Moreover, various filter media (FFP2 mask, *e*PM_1_ 85%, and *e*PM_1_ 55%) exhibit different effects. The difference between OME and ME < 0.3 µm increases when air filter efficiency decreases.

Table 1 shows, for each filter media, the GSD sensitivity analysis results of the OME and the mass efficiency (ME) for particles below 0.3 µm (equivalent diameter), as several authors usually assume for PM_0.3_ efficiency^13–19^. For better visualization, Fig. 3 summarizes the data in Table 1.

**Table 1:** GSD sensitivity analysis results for different definitions of mass efficiency

	GSD 1.25	GSD 1.86	GSD 2.20	GSD 3.00
*Generated aerosol*				
CMD [nm]	75	75	75	75
MMD [nm]	87	238	484	2803
GSD	1.25	1.86	2.20	3.00
Cumulative mass concentration [mg m^−3^]	20	20	20	20
*FFP2 mask*				
MPPS of filter [nm]	65			
Efficiency at MPPS	94.5%			
Overall mass efficiency	96.4%	98.5%	99.0%	99.7%
Mass efficiency for particles <0.3 µm	96.4%	98.2%	98.4%	98.5%
*ePM*_*1*_ *85% filter*				
MPPS of filter [nm]	134			
Efficiency at MPPS	60.0%			
Overall mass efficiency	67.1%	70.0%	81.6%	96.7%
Mass efficiency for particles <0.3 µm	67.1%	64.6%	65.1%	65.6%
*ePM*_*1*_ *55% filter*				
MPPS of filter [nm]	110			
Efficiency at MPPS	11.0%			
Overall mass efficiency	12.0%	19.9%	37.0%	77.6%
Mass efficiency for particles <0.3 µm	12.0%	14.4%	15.3%	15.9%

GSD, MMD, and CMD are linked by the equation MMD = CMD exp(3 ln^2^ GSD) for a lognormal distribution^5^. Increasing MMD or the dispersion of the test aerosols tends to overestimate the OME, driven by a higher concentration of larger particles that are easily captured. However, this phenomenon is less evident for filter media with high efficiency, like FFP2 masks. For instance, when the efficiency is close to 100% at MPPS, shifting the particle size distribution towards larger will not increase the efficiency appreciably. The underlying conceptual mistake remains hidden since the lowest efficiency is already nearly 100%. The same does not occur when testing filters with lower efficiency (*e*PM_1_ 85% or *e*PM_1_ 55%).

As expected from air filtration theory, the results show that increasing the aerosol dispersion increases the OME due to a higher mass concentration of larger particles (right tail of the lognormal distribution) which are easily captured. This effect is less relevant for high-efficiency filter media, such as the one used for FFP2 masks. Suppose the efficiency at the MPPS, i.e., the particle size corresponding to the maximum penetration, is nearly 100%. In that case, the shift toward particles easier to capture will not increase the efficiency because the lowest efficiency is already close to 100%.

This effect masks the conceptual mistake.

However, when measuring the performance of other standard filter media, the overall efficiency drastically changes. We show two relevant examples: *e*PM_1_ 85% and *e*PM_1_ 55% according to ISO 16890-1:2016^29^. For *e*PM_1_ 85%, as the GSD increases from 1.25 to 3, the OME increases from 67.1% to 96.7%, while it increases from 12.0% to 77.6% for *e*PM_1_ 55%.

The overall efficiency approach does not provide insight into how the removal efficiency changes with particle size. One can use it to check the conformity requirements against a minimum efficiency or a maximum penetration, like for face masks. One can use it to compare the filtration performance of a new material against a benchmark or to evaluate the stability of filter media production quality. However, the particle size distribution of the test aerosol challenging the filter must be strictly fixed and well-known. Otherwise, researchers cannot compare the OME data obtained with different particle size distributions, as they are misleading or meaningless^13,19,30–32^.

In addition, OME cannot provide removal efficiency values at the MPPS, as shown in Table 1. We note that the values shown for the MPPS also depend on the airflow rate. The confusion between the efficiency at MPPS and at 0.3 µm comes probably from the definition of HEPA filter, proposed during the Second World War when Irvin Langmuir developed the hot DOP (Dioctyl-phthalate) test^33^ and described the challenge aerosol particles distribution with an MMD at 0.3 µm^34^. See also the following section’s discussion about the US Military Standard 282.

ISO 29463-3:2011^35^ shows how to determine the MPPS on a flat media sheet and summarizes helpful information. The MPPS of most non-charged filter media is between 0.1 and 0.2 µm. The MPPS of charged filter media is much smaller, sometimes down to 40 nm. Hence, PM_0.3_ has nothing to do with the efficiency in correspondence to the MPPS. In general, measuring the overall efficiency will not provide the efficiency corresponding to a specific size unless the test aerosol is strictly monodisperse. In practice, air filtration test methods do not adopt this approach unless for calibration purposes with reference polystyrene PSL microspheres.

We also note that OME and PM_0.3_ are intrinsically different. OME considers all the particle sizes in the test aerosol challenging the filter, while PM_0.3_ data refer only to particles below the 0.3 µm threshold. When the test equipment cannot distinguish the particle size because the particle sensing instrument is a photometer, it will not provide the PM_0.3_ information unless all the particles of the challenging aerosol are smaller than 0.3 µm. Such a condition is impractical.

Finally, testing air filters and using a test aerosol with an MMD corresponding to a specific size does not imply obtaining the efficiency corresponding to that size or to the fraction of particles below that size. In fact, in the particle size distribution with a certain MMD, particles larger than the MMD contribute 50% of the mass to that size distribution.

## Interpretation of the data provided by particle photometers vs. particle spectrometers

Optical instruments for measuring particle concentration are the most common particle-sensing instruments to assess air filtration performance. Light scattering is the physical phenomenon that correlates particles’ presence and size by detecting the scattered light’s intensity and angle. By solving Maxwell’s equations, the Mie theory calculates the scattering and absorption of light by a single spherical particle. The result is a function of the size, the refractive index, and the wavelength of the incident light. Consequently, the amount of scattered light is also a function of the type of light source and the particle’s chemical composition.

By analyzing one particle at a time, optical particle spectrometers generate a correlation between the intensity of the scattered signal and a single particle, enabling the determination of the particle’s size and, consequently, its volume and mass (see schematics of Fig. 1).

On the other hand, particle photometers analyze a group of particles simultaneously and do not allow for creating a correlation between the intensity signal and single particles. Instead, they generate a single signal for a group of particles that, in the case of a polydisperse aerosol, results from the interaction of the light source with a size range of particles that may be wide. This type of instrument correlates the amount of scattered light with the total particles’ volume, i.e., their total mass if their density is known. Photometers need calibration against the gravimetric measurement of aerosols of known size. The measurement accuracy of a photometer depends on whether the properties of the measured aerosol differ from those of the calibration aerosol. Indeed, most of the time, test rig manufacturers carry out suitable calibration procedures considering specific applications.

Using photometers cannot provide data referring to a specific particle size unless using a monodisperse aerosol. Hence, a test rig using photometers and a polydisperse aerosol cannot measure the filtration efficiency at the MPPS. These data are often crucial for assessing high-efficiency filter media.

In several countries, many assume that the efficiency of HEPA filters is measured at 0.3 μm particle size according to the US Military Standard 282^36^. Several experts include 0.3 μm in the definition of HEPA filter. However, 0.3 μm is the mass mean diameter of the aerosol specified by the US Military Standard 282, while the count mean diameter of this aerosol is around 0.18 μm (ISO 29463-2^25^). Hence, testing a filter using an aerosol with a count mean diameter of 0.3 μm overestimates its efficiency. To avoid similar errors, one shall consider the complete particle size distribution, not its mean properties.

**Fig. 4:**
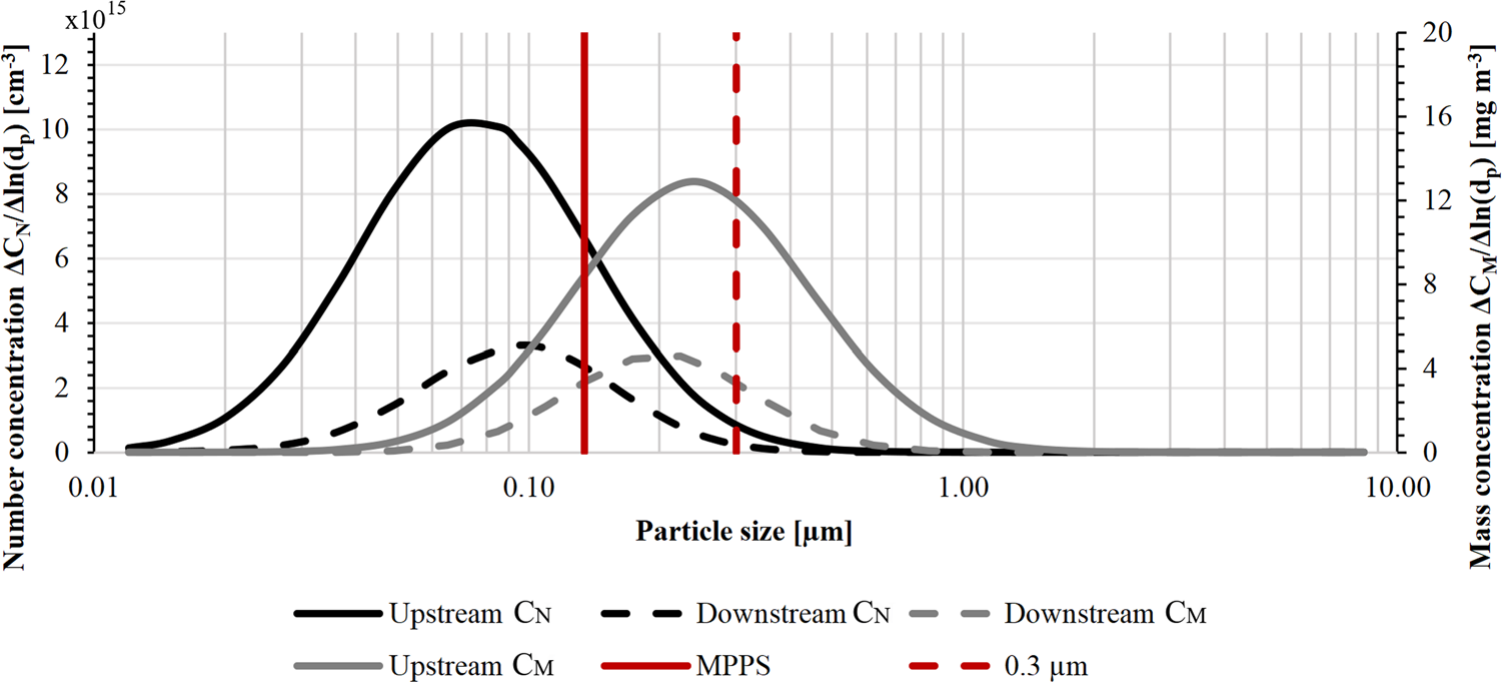
Difference between the MPPS and the mass median diameter. The graph shows the distribution of count concentration and mass concentrations (respectively, Δ*C*_*N*_/Δln(*d*_*p*_) and Δ*C*_*M*_/Δln(*d*_*p*_), where *d*_*p*_ is the particle diameter) as a function of particle size of a test aerosol with a lognormal distribution. Solid lines represent the distributions of upstream number concentration (black) and mass concentration (gray), while dashed lines represent downstream distributions of an *e*PM_1_ 85% filter. The vertical red lines indicate the distinct positions of the MPPS (solid line) and the mass median diameter (dashed line) at 0.3 µm. The MPPS is smaller than the mass median diameter, resulting in a higher efficiency value at 0.3 µm compared to the value at MPPS.

Figure 4 shows the count and mass distribution as a function of particle size of a test aerosol challenging an *e*PM_1_ 85%^29^ filter. We note the clear difference between the mean values of the two distributions and the MPPS position in this specific case. The MPPS changes with the filtration velocity.

## Concluding remarks and recommendations

Despite the growing concern about the health effects of exposure to the so-called PM_1_, PM_0.3_, or PM_0.1_, no established air quality standard exists for their definition. We discussed the implications of using these generic terms without accurately defining their meaning and showed their impact on the data describing air filtration performance. Table 2 suggests approaches to designing a correct experimental assessment so that researchers can compare new media and technology to existing ones with better reliability.

**Table 2:** Overview of key points of concern when evaluating the filtration performance of novel filter media and recommendations to address them

Points of Concern	Recommendations
Use of the term PMxx	• Do not use the term PMxx to define an aerosol fraction below a specific size without specifying sampling, measurement, and experimental protocols in detail. • Remember their meaning when using PM_10_ and PM_2.5_ terms and relate them to reference methods. • Use aerodynamic and physical size within the appropriate range. • Report the particle size distribution used for the experiment, indicating how it was measured.
Approach for measuring air filter efficiency	• Provide detailed information about the physical and chemical nature of the test aerosol. • The best methodology to understand and demonstrate fibrous filtration performance is measuring the efficiency as a function of the particle size. • Particle spectrometers can provide efficiency as a function of the particle size with a single test. • Photometers require challenging the filter with a monodisperse aerosol and measuring the reduction in particle concentration for one specific size. The procedure requires multiple tests as the investigated particle sizes. • If researchers are measuring a fixed polydisperse aerosol’s total particle number or mass, they must avoid expressing efficiency for specific PM fractions or at the MPPS. In this case, researchers can only compare filtration efficiency with benchmark media. • If possible, use a standardized test method following its qualification procedures.
Expressing air filter efficiency	• Air filtration efficiency as a function of particle size is necessary for calculating the removed PMxx fraction. • To calculate the air filter efficiency for a specific PMxx fraction, use the procedure explained in ISO 16890-1. • Expressing air filtration efficiency with a single number (e.g., 90%) requires a detailed test aerosol characterization to ensure meaningfulness and reproducibility.
Use of the photometer	• A photometer measurement using a polydisperse test aerosol cannot provide the efficiency corresponding to or below a specific particle size. • Single number efficiency obtained with a photometer is extremely sensitive to large particles in the tail of the particle size distribution. • Comparison among different filter media is valid only if the upstream distribution is identical.
Identification of MPPS and corresponding efficiency	• A photometer measurement using a polydisperse challenge aerosol cannot identify the MPPS. • Do not combine a photometer with a polydisperse aerosol having an MMD equal to the MPPS to obtain the maximum penetration. • When using a spectrometer, the upstream distribution must cover the particle size range where the MPPS falls. • Associate the efficiency at the MPPS with the media velocity because the MPPS is a function of the airflow rate.
